# A robotic model of efficient prey finding in the gleaning bat *Micronycteris microtis*

**DOI:** 10.1242/jeb.250818

**Published:** 2026-01-14

**Authors:** Dieter Vanderelst, Inga Geipel, Herbert Peremans

**Affiliations:** ^1^ Departments of Biological Sciences, Electrical & Computer Engineering, and Mechanical & Materials Engineering, University of Cincinnati, Cincinnati, OH 45221, USA; ^2^ Smithsonian Tropical Research Institute, Luis Clement Avenue, Bldg. 401 Tupper, Ancon, Panama 0843-03092, Republic of Panama; ^3^ Department of Engineering Management, University of Antwerp, 2000 Antwerpen, Belgium

**Keywords:** Echolocation, Foraging, Sensory ecology, Bio-robotics

## Abstract

The common big-eared bat (*Micronycteris microtis*) gleans its prey directly from plant surfaces. Previous experiments have shown that this bat exploits the specular reflection effect to discriminate between occupied and empty leaves. Using this effect requires the bat to position itself such that it can ensonify the leaves at sharp angles. If the bat can perceive the orientation and position of individual leaves, it could position itself to take advantage of the specular reflection. However, this would be a highly inefficient foraging strategy. The bat would first have to inspect each leaf to determine its position and orientation. Given that, under natural conditions, the vast majority of leaves do not feature prey, this would require the bat to spend most of its time collecting information on empty leaves. Here, we propose a strategy that allows a bat to exploit the specular reflection effect without inferring the position and orientation of individual leaves. We implement this strategy on a robotic arm equipped with a sonar head. The robot is tasked with finding a 3D-printed dragonfly on one of a set of artificial leaves. The robot follows an echo amplitude gradient and abandons this search whenever the echoes become too weak. Importantly, the robot does not actively find or locate individual leaves. We show that the proposed sensorimotor model can exploit the specular reflection effect to efficiently and effectively locate prey. Our results increase the plausibility of *M. microtis* using the specular reflection effect under natural conditions.

## Teaser

**Highlighted Article:** A simple sensorimotor strategy, using echo amplitude and interaural level differences, enables an efficient gleaning foraging strategy in cluttered environments, mimicking the foraging behavior of echolocating bats without requiring explicit knowledge of leaf position or orientation.

## INTRODUCTION

Echolocating insectivorous bats often hunt in open airspace, where prey echoes are clearly separated from distracting clutter echoes (Denzinger and Schnitzler, 2013). In contrast, gleaning bats feed on prey or food items found on surfaces such as leaves, trunks or the ground. Passive gleaners rely on prey-generated sounds, such as rustling noises, to locate their targets (Arlettaz et al., 2001). Active gleaners, in contrast, use echolocation to forage. Most active gleaners forage for fruits and flowers. Many plants depend on bats for pollination or seed dispersal and therefore advertise their presence to these bats through specific echo properties of their inflorescences (Simon et al., 2011; Schnitzler and Denzinger, 2024) and by positioning themselves prominently against their background (Von Helversen and Von Helversen, 2003). However, some species, such as the common big-eared bat (*Micronycteris microtis*), have adapted to glean insect prey directly from plant surfaces using only echolocation (Swift and Racey, 2002; Denzinger and Schnitzler, 2013; Kalka and Kalko, 2006; Denzinger et al., 2018). Unlike passive gleaners that rely on prey-generated sounds or active gleaners attracted to acoustically conspicuous plants, these bats must detect insect prey that have not evolved to stand out acoustically. This ability represents a rare and challenging foraging strategy among the nearly 1200 echolocating bat species (Schnitzler and Denzinger, 2024; Jones, 2013).

The active gleaner *M. microtis* has been observed foraging for prey perched on vegetation within the dense rainforest understory, where prey echoes are hidden in background clutter echoes. Therefore, it is likely to have developed strategies to locate its prey effectively in this environment. Geipel and co-workers report experiments in which *M. microtis* successfully captured prey from broad-leafed plants (Geipel et al., 2013) and from a single artificial leaf (Geipel et al., 2019; Geipel, 2007). These authors suggested that *M. microtis* finds prey by exploiting the so-called specular reflection effect (SRE) (for a related hypothesis, see Denzinger and Schnitzler, 2013; Swift and Racey, 2002), a strategy also used by trawling bats taking prey from the smooth surfaces of bodies of water (Siemers et al., 2005). The SRE refers to an acoustic effect in which smooth surfaces reflect most of the sound energy away from the emitter at large incidence angles. Consequently, the bat could distinguish empty and occupied leaves by positioning itself to ensonify them at a sufficiently large angle. Most of the energy will be reflected away for unoccupied leaves, producing a weak echo. In contrast, when prey occupies the leaf, more of the sound is scattered back toward the bat, resulting in a stronger echo.

**Fig. 1.**
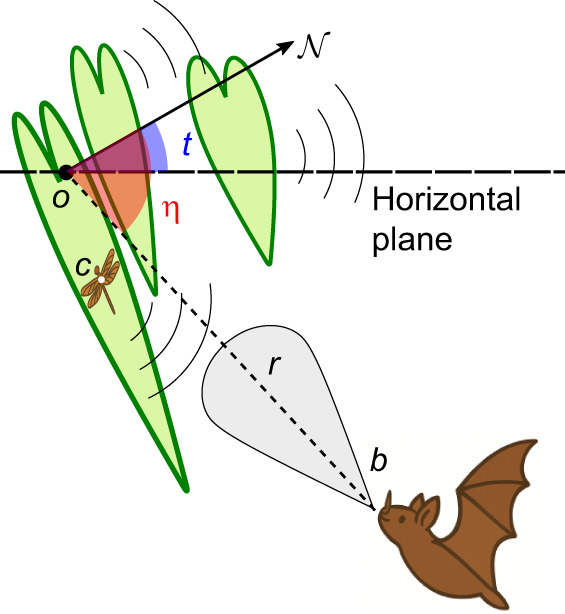
**Echolocating agent exploiting the specular reflection effect (SRE) in detecting insect prey perched on one of several leaves.** The location *o* is the point on the leaf's surface where the sonar beam intersects it; *b* and *c* denote the echolocating agent's and insect prey's positions, respectively. The vector 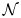 denotes the leaf's normal. The angle of incidence η (indicated in red) is the angle between the vector 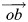 and the leaf's normal 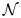. The angle between the leaf normal and the horizontal plane is the leaf tilt *t* (indicated in blue).

To support this hypothesis, Geipel et al. (2019) ensonified a leaf with and without insect prey (i.e. a dragonfly). Their results showed that for large incidence angles, i.e. large angles between the sonar beam and normal to the leaf (see Fig. 1 for a depiction of the geometry of this foraging situation), empty leaves result in weak echoes as they reflect most of the acoustic energy away from the bat. In contrast, leaves occupied by prey reflect stronger echoes toward the bat over a much wider range of incidence angles. In the accompanying behavioral experiment (Geipel et al., 2019), *M. microtis* ensonified a leaf containing prey using incidence angles that were shown to increase the difference in echo amplitude between empty and occupied leaves (Geipel, 2007; Geipel et al., 2013).

The SRE hypothesis suggests that differences in echo strength can serve as an acoustic cue, allowing bats to discriminate between occupied and empty leaves. However, as mentioned, to take advantage of this cue, the bat must ensonify the leaves at favorable incidence angles. Specifically, the bat needs to position itself so that it can ensonify the leaf at a large incidence angle. At such angles, the SRE ensures that most of the acoustic energy is reflected away from the bat when the leaf is empty, resulting in a weaker echo that can be distinguished from a stronger echo when the leaf is occupied.

If we assume that the bat is capable of perceiving the orientation and position of the leaf, it could easily position itself to take advantage of the SRE, as *M. microtis* is capable of hovering (Geipel, 2007; Geipel et al., 2013). However, this assumption would imply a highly inefficient foraging strategy. The bat would first have to inspect each leaf to determine its position and orientation before positioning itself appropriately and exploiting the SRE to assess whether it contains prey. Under natural conditions, the vast majority of leaves do not feature prey. Therefore, this would require spending most of its time collecting information on empty leaves.

Maximizing foraging efficiency requires a strategy that quickly rejects or ignores empty leaves while directing the bat toward leaves with prey. We propose a strategy that allows the bat to exploit the SRE without inferring the position and orientation of individual leaves. The rationale is that empty leaves act as specular (mirror-like) reflectors, which produce echoes whose amplitudes vary sharply with changes in incidence angle (Geipel et al., 2019). However, rather than explicitly detecting this variability, the bat can exploit a simpler consequence of it: as the bat moves, the incidence angle will eventually reach a point where the echo becomes weak, falling below a threshold and prompting abandonment of the approach. In contrast, leaves occupied by prey reflect sound more diffusely, leading to a consistently detectable stream of echoes throughout the approach. This mechanism allows for efficient prey detection without requiring the bat to infer the structure or location of individual leaves.

Following a biorobotic approach (Webb, 2000, 2001; Gravish and Lauder, 2018), the proposed strategy is implemented and tested as an explicit sensorimotor model that controls a robotic echolocating agent. In contrast to a computer model, a robotic approach does not require modeling the interaction of sound with complex environments, which remains challenging and can only be approximated in simulation. In addition to this methodological reason for experimenting with a robotic implementation of the foraging strategy, a more fundamental reason is our hypothesis that a significant part of the intelligence required to solve this task is not located in the bat itself, but rather in the interactions between an echolocating agent and its environment, seen as coupled dynamical systems (Beer, 1997; Warren, 2006; Brooks, 1999; Di Paolo et al., 2017).

The proposed sensorimotor model builds on our previous robotic work, where we introduced a simple sensorimotor model that used binaural intensity signals to home in while foraging (Nguyen and Vanderelst, 2022, 2024), including on prey perched on a leaf (Nguyen et al., 2021). Here, we extend this earlier model with an SRE-based mechanism designed to be attracted to stable reflectors while ignoring or abandoning erratic, specular reflectors. This mechanism functions as a behavioral matched filter, which emphasizes relevant sensory stimuli while suppressing distracting ones. In its original form, the matched filter concept proposed by Wehner (1987) aimed to limit the information processed by the brain, freeing it from the need for complex computations. More recently, Warrant (2016) reframed sensory matched filters as mechanisms to minimize the energy required to solve a task. Using this energy-efficiency criterion, we propose the concept of a behavioral matched filter as a generalization of the sensory matched filter. The efficient foraging strategy described here constitutes such a behavioral matched filter. Specifically, the dynamics of the echolocating agent, when coupled with its environment, naturally lead the agent to respond only to task-relevant stimuli (leaves with prey) while ignoring distracting stimuli (empty leaves).

List of symbols and abbreviations

*b*: position of echolocation agent
*c*: position of insect prey position on leaf
*d*: characteristic size of an object
*d*_0_: distance estimated from the arrival time *t*_0_
*D*_min_: minimum distance between the current positions *X*_0_,*Y*_0_,*Z*_0_ and previously stored positions from which the robot previously (attempted) to capture prey
*I*_L_, *I*_R_: signal envelopes integrated over a fixed window around *t*_0_ for the left and right receivers in the sonar head
ILD: interaural level difference
*k*: angular wave number, defined as *k*=2π/λ
L_1_ to L_5_: labels associated with each of the 5 leaves in both setups
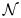
leaf normal
*o*: point on leaf surface where the center of the sonar beam intersects
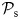
pitch of the sonar head, i.e. rotation of the sonar head around axis *Y*_s_
SRE: specular reflection effect
*t*: leaf tilt angle, the angle between the leaf normal 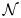 and the horizontal plane
*t*_0_: arrival time of the first echo above threshold
*X*_0_,*Y*_0_,*Z*_0_: *X*, *Y* and *Z* positions of the estimated location from which the echo returns
*X*_l_,*Y*_l_,*Z*_l_: *X*, *Y* and *Z* positions of the leaf
*X*_s_,*Y*_s_,*Z*_s_: *X*, *Y* and *Z* positions of the robot sonar head
*X*_w_,*Y*_w_,*Z*_w_: *X*, *Y* and *Z* positions of the robot in world coordinates
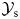
yaw of the sonar head, i.e. rotation of the sonar head around axis *Z*_s_
α_0_: azimuthal origin of the first echo as estimated using a lookup table
Δ*I*: difference between *I*_L_ and *I*_R_ on a logarithmic scale (Eqn 5)
η: incidence angle, i.e. angle between the vector 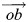 and the leaf normal 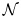
λ: wavelength (m)

## MATERIALS AND METHODS

We modeled the echolocating bat using an artificial echolocating agent, i.e. a robot arm mounted on a 3-m-long linear track, instrumented with a binaural sonar head. In each experiment, the agent was presented with multiple artificial leaves with a 3D-printed prey item perched on one of the leaves. We assumed that the prey is sitting in the center of the leaf, because Geipel et al. (2019) presented the ensonification data for this case only. The agent's task is to approach the one leaf with a prey item sitting on it while disregarding the surrounding empty leaves.

### Robot setup

**Fig. 2.**
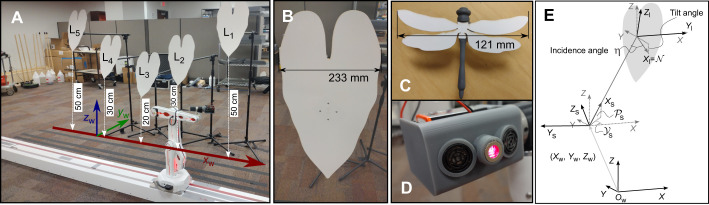
**Overview of the first experimental setup.** (A) Image showing the robot arm mounted on the track. The world coordinate frame is overlaid. Note that the horizontal plane (*Z*=0) does not coincide with the floor of the room but intersects the robot's elbow joint. Vertical white arrows indicate the height of the lower tip of each leaf above the horizontal plane. (B) Detailed view of one of the leaves. The four dots are the heads of 4 M2 screws used to hold the leaf in place. (C) View of the 3D-printed dragonfly that was used as a prey item. (D) View of the sonar head showing the two MB1360 sonar sensors and the laser diode. (E) Relationship between the different coordinate systems associated with the world (subscript w), sonar (subscript s) and leaf (subscript l). These coordinate systems apply to both experimental setups reported in this paper. The sonar beam is pointing along the *X*_s_-axis. Note that the *X*_l_-axis coincides with the normal 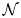 to the leaf. The *Z*_l_-axis coincides with the longitudinal direction, and the *Y*_l_-axis with the transverse direction of the leaf. The tilt angle of the leaf, as referred to in the text, corresponds to minus the pitch angle around the *Y*_l_-axis. The incidence angle η denotes the angle between the direction of the sonar beam and the normal to the leaf.

We instrumented an R12 robot arm (ST robotics, Princeton, NJ, USA) with a sonar system (see Fig. 2A–D). The robot arm was mounted on a 3-m-long track, aligned with the *X*-axis of the world coordinate frame. In the *Y* and *Z* directions (with *Z*=0 at the shoulder joint of the robot arm), the robot had a reach of approximately ±50 cm. The robot arm allowed translation and rotation of the sonar head (Fig. 2D).

Pitching the sonar head rotated it around the sonar head's *Y*_s_-axis. Yaw 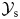 was the angle around the sonar's *Z*_s_-axis. See Fig. 2E for the definition of the different coordinate systems used. To verify the direction of view of the sonar head system, a laser diode was mounted in the head between two sonar rangefinders (MB1360, Maxbotix, Brainerd, MN, USA; Fig. 2D). The directionality of the emitters was quantified and compared with the simulated directionality of *M. microtis* of Vanderelst et al. (2010) in Fig. S1.

The sonar sensors were mounted in a 3D-printed case at a 30 deg angle between their acoustic axes, with a microcontroller controlling their pulsing sequence. Each sensor was pulsed in succession with a 0.5 s delay between pulses to prevent interference. The sonar sensors recorded the resulting echo envelope. This envelope, an amplitude-modulated signal with lower frequency content than the underlying 42 kHz carrier wave, was wirelessly relayed to the computer controlling the robot arm. Unlike sampling the carrier wave, which would require a rate of at least 84 kHz to satisfy the Nyquist criterion, the lower frequency content of the envelope allowed for the much lower sampling rate of 10 kHz.

### Experimental setup 1

In the first of two setups used in this paper, we placed five heart-shaped leaves made of thin cardboard along the robot's track (*X*-axis; see Fig. 2A). The direction of the track is the direction in which the robot has the most extensive reach. Therefore, by placing the leaves along the track, our robot could inspect and visit multiple leaves. We refer to individual leaves as L_1_ through L_5_.

Because the impedance difference between air and solids is large, ultrasonic echoes are dominated by geometry rather than material properties. This is demonstrated, for example, by boundary-element simulations of bat head-related transfer functions that closely match measurements without accounting for the material properties of the bat's head and pinnae (e.g. De Mey et al., 2008). Moreover, behavioral and ensonification studies have also employed artificial leaves (Geipel et al., 2013, 2019). Thus, geometry-matched paper artificial leaves are functionally equivalent for the questions studied here.

The leaves were mounted at different heights above the floor on microphone booms using a rotational joint between the back of the leaf and the boom, allowing us to vary the tilt angle of the leaves to investigate how the proposed strategy performs in the face of differently oriented leaves. In this paper, a leaf tilt angle of 0 deg implies that the leaf is oriented perpendicular to the floor. For negative tilt angles, the leaf is tilted backward (that is, the normal of the leaf is pointing upward). Positive tilt angles mean that the leaf is rotated forward (i.e. the normal points toward the floor; see also Fig. 2E). We used the following tilt angles for the leaves: 10, 0, −10, −20, −30 and −40 deg. The reason we selected one angle that resulted in forward tilted leaves and four that resulted in backward tilted leaves is that we model a bat looking up at leaves to exploit the SRE. In the Discussion, we further explain the geometric constraints a bat needs to satisfy in order to exploit the SRE. This will clarify that a symmetric situation in which a bat looks down at forward tilted leaves is also possible. However, as the bats in the experiments of Geipel (2007) and Geipel et al. (2019) looked up at backward tilted leaves (i.e. negative tilt angles), that is the situation we focus on.

Although the sonar sensors used in this paper employ narrowband sonar signals around 42 kHz, *M. microtis* uses broadband calls with a frequency range of approximately 70 to 140 kHz (Geipel et al., 2013). Taking the center frequency of 100 kHz as a conservative reference, the wavelengths used by our robot are more than twice as long as those used by *M. microtis*. To compensate for differences in frequencies used by *M. microtis* and our robot, we scaled all distances and dimensions in our setup by a (conservative) factor of 2.

This scaling is valid because, in acoustics, the interaction between sound waves and objects is determined by the ratio of the wavelength (λ) to the size of the object (*d*), rather than their absolute values. This ratio governs key phenomena such as reflection, scattering and diffraction. By scaling both the wavelength and the size of the object proportionally, we preserve the dimensionless parameter *d*/λ (or equivalently *k*·*d*, where *k*=2π/λ, which ensures that the wave–object interactions remain unchanged.

The shape of the leaves (Fig. 2B) was traced as closely as possible from the leaf used for the experiments reported by Geipel et al. (2019; their fig. 3). We scaled the resulting shape, which resulted in a leaf 405 mm tall and 233 mm wide.

**Fig. 3.**
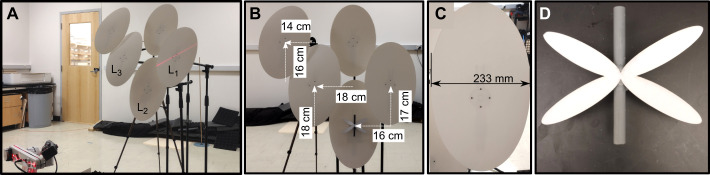
**Overview of the second experimental setup.** (A) Image showing an overview of the setup with the three leaves used labeled. (B) Front view of the leaves with horizontal and vertical distances between the centers of the leaves marked by arrows. (C) A detail of one of the leaves. (D) A view of the stylized dragonfly.

We placed a naturalistically shaped, 3D-printed dragonfly in the center of one of the leaves during each trial (Fig. 2C). This prey shape was selected because dragonflies are a part of the natural diet of *M. microtis* (Geipel et al., 2013). Indeed, Santana et al. (2011) observed bats in Panama and found dragonflies to be one of the most common prey items taken by *M. microtis*. Moreover, in behavioral experiments, dragonflies were one of the prey items offered to *M. microtis* (Geipel, 2007; Geipel et al., 2013, 2019). The shape of the artificial dragonfly was obtained by digitizing the dorsal view of the dragonfly *Cordulegaster annulatus* (Editor of Naturalists’ Gazette, 1890). It was then scaled to a length of approximately 9 cm, double the body length of the prey items used by Geipel et al. (2019).

The dragonfly's body was 3D printed on a Form 3L resin printer (Formlabs, Somerville, MA, USA) using Grey Resin FLGPGR04 (Formlabs, Somerville, MA, USA). The wings were cut from the same thin cardboard used for the leaves with a Glowforge laser cutter (Glowforge, Seattle, WA, USA) and glued to the center of the printed body (Fig. 2C).

To replicate the mounting conditions described in Geipel et al. (2019), where the dragonfly protruded 2 cm from the leaf surface, we added a foot to the 3D-printed dragonfly that placed it 4 cm above the leaf. A magnet was embedded in the dragonfly's foot, allowing it to be easily mounted and removed from the cardboard leaves, which had a corresponding magnet in their center at the back.

### Experimental setup 2

We also built a second setup to ascertain whether the results obtained with the first experimental setup can be generalized. The second experimental setup used in this paper is shown in Fig. 3. In this setup, the artificial leaves were spaced much closer together. In fact, there was some overlap between the leaves. In addition, the shape of the leaves was stylized as an oval with the same surface area, maximum width and maximum height as the leaves used in the first setup. By stylizing the leaves and creating some overlap between them, we wanted to test whether the results depend on the precise shape of the leaf and whether they are robust against overlap between the leaves.

In addition to stylizing the leaf, we simplified the shape of the 3D-printed dragonfly. Its body was simplified to a cylinder with the same length and volume as the naturalistic dragonfly shown in Fig. 2C. The wings were cut from the same material as the leaves and simplified into four overlapping ovals with a total surface area matching that of the naturalistic dragonfly's wings. Again, this was done to test the sensitivity of the results to the precise shape of the target.

The leaves in this second setup were only tested at a tilt angle of −30 deg. In addition, we only placed a dragonfly on three out of five leaves. The two highest leaves were out of reach of the robot. In this setup, these artificial leaves only provided potentially distracting echoes.

### Robot controller

Before describing the robot controller, that is, the proposed and tested sensorimotor control strategy that would allow *M. microtis* to home in on prey without inferring each leaf's position and orientation, in detail, we briefly summarize the robot's behavior to clarify its rationale.

#### Overview of the sensorimotor strategy

The details of the robotic controller are presented in the next section. Here, we summarize the robot's behavior. The robot arm was programmed to run the proposed sensorimotor model, starting at one end of the track and progressing along the row of leaves while trying to find the location of the artificial dragonfly. The robot initially moves semi-randomly through space while performing sonar measurements at a fixed rate. We call this behavior ‘random exploration’.

Whenever the robot receives a sufficiently strong echo in at least one ear, it attempts to locate the source of the echo. This is done by minimizing the interaural level difference (ILD) by horizontally rotating the sonar head (yaw rotation). When the ILD is small enough, the sonar head is also translated in the resulting direction. This behavior is called ‘echo following’. Intermittently, the robot also tries to maximize the overall echo amplitude by performing a ‘pitch correction’. If the echo amplitude falls below a threshold, the robot reverts to random exploration.

In sum, the robot attempts to climb an echo-amplitude gradient, abandoning this strategy whenever the echoes become too faint. Importantly, echo amplitude and ILD are the only cues available to the robot; it does not explicitly detect or identify leaves. This sensorimotor strategy directly builds on the rationale introduced earlier: empty leaves, acting as specular reflectors, produce echoes with highly variable amplitude as the angle of incidence changes during approach. In contrast, prey-bearing leaves generate more diffuse reflections, resulting in a more stable and consistently detectable echo. These stable echoes provide a reliable acoustic cue that the robot can follow to reach a leaf. In contrast, the echo from an empty leaf is likely to drop below the threshold at some point during the approach, causing the robot to abandon that trajectory.

If the robot received an echo from less than 50 cm, the program paused, and the experimenter evaluated where the robot was looking using the built-in sonar head laser. If the laser beam intersected the leaf with the dragonfly on it, the latter was removed to simulate a capture. In case the robot's laser beam intersected a leaf without a dragonfly or no leaf at all, this was noted. The robot then resumed the trial. The trial ended when the robot reached the end of the track. The robot's behavior, as driven by the proposed sensorimotor model, is illustrated in several videos (see below for a link to this material).

The stopping distance of 50 cm was chosen to address the technical limitations of our sonar system. Unlike the bat's calls, which are short and broadband, our sensor emits sonar signals with a duration of approximately 2 ms at a fixed amplitude. At closer distances, this prolonged emission leads to a significant overlap between the outgoing signal and the returning echo, making accurate echo detection difficult.

At the same time, it is important to note that this stopping distance still allows us to model the relevant decision-making distances for the bat. Because we scaled our setup by a factor of 2, the 50 cm stopping distance corresponds to 25 cm in the bat's original scale. This distance aligns with the findings of Geipel et al. (2019), who documented that *M. microtis* begins its final approach and has already decided which leaf to target at distances shorter than approximately 25 cm. These data are shown in Fig. S2. Therefore, by setting the stopping distance to 50 cm, we compensate for the sonar system's limitations while still modeling the distances at which the bat is actively deciding which leaves to land on.

To help the reader understand the operation of the robot, we have created dynamic visualizations that replay all trials reported in this paper. They show the location of the robot with respect to the leaves, the acoustic signals in both ears, and the decisions made by the robot. A screenshot of one of the videos is provided as Fig. S3A. The full videos are available in the repository with supporting data (https://doi.org/10.17605/OSF.IO/AC4VW). The repository also contains videos showing five representative trials (see Fig. S3B for a screenshot). In addition to these videos, the repository contains all the data and code for this paper.

#### Details of the sensorimotor strategy

**Fig. 4.**
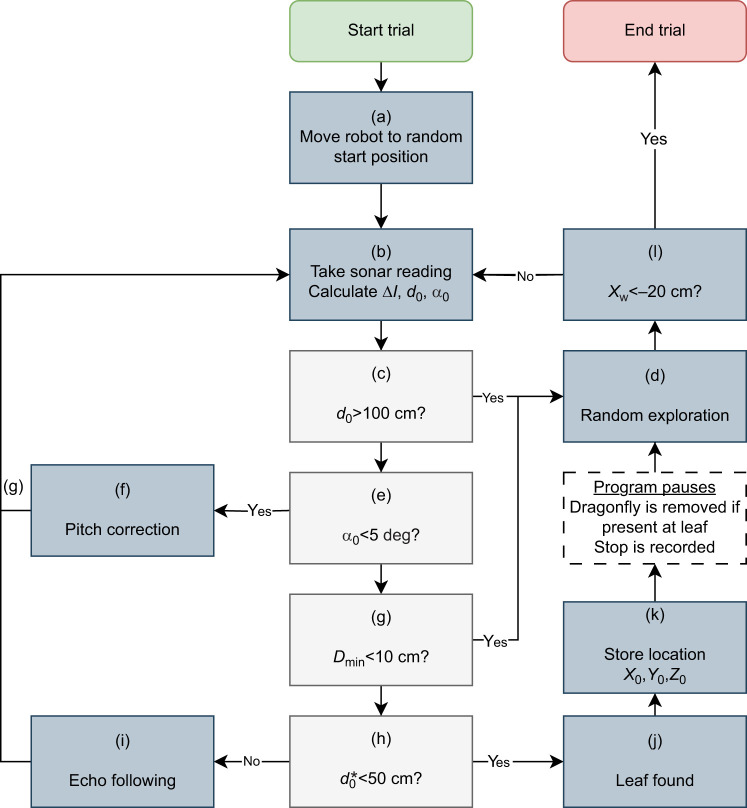
**Flowchart of the sensorimotor model for each trial.** The variables *X*_w_, Δ*I*, *d*_0_, *D*_min_ and α_0_, as well as the different actions executed by the robot, are described in the Materials and Methods.

This section describes in detail the sensorimotor model controlling the robot as it attempts to find leaves occupied by an artificial dragonfly. Throughout this section, we refer to the flow chart in Fig. 4 and the labels therein.

Each trial started by moving the sonar head to the starting position. The starting position *X*_w_ was set to 120 cm. Other pose parameters were selected at random from the following intervals (Fig. 4, label a):
(1)

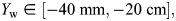

(2)

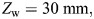

(3)

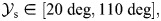

(4)

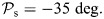


At this position, in both setups, L_1_ is located to the left of the robot from the perspective of the sonar head. The vertical position of the robot, *Z*_w_, aligns with the lower tips of L_2_ and L_4_ in the first setup and with L_2_ in the second setup. The range of yaw angles (Eqn 3) ensures that the sonar head is oriented in the general direction of the row of leaves.

At the beginning of each trial, a 3D-printed dragonfly was mounted on one of the leaves (L_1_ to L_5_ in the first setup, L_1_ to L_3_ in the second setup), while the remaining leaves were left empty. Starting from this initial position, the robot followed a sequence of steps as outlined in Fig. 4.

First, the robot collected an echo measurement by pulsing each sonar sensor in succession and recording the returned echo envelopes (Fig. 4, label b). The resulting echo envelopes were thresholded. The threshold was hand-picked but not optimized. A range of values could have been selected for this threshold. In the case of the bat, this threshold could be learned through experience or adapted dynamically based on the environmental context. Moreover, bats could use a more complex decision criterion based on multiple acoustic cues. However, and crucially, our data presented below show that a simple decision criterion based on amplitude can already result in good performance.

The first sample above the threshold, across both envelopes, was considered as the arrival time *t*_0_, resulting in the corresponding estimated distance *d*_0_ of the first echo. Both signal envelopes were integrated over a fixed window around *t*_0_, resulting in *I*_L_ and *I*_R_ (see Fig. S4). A brief integration window of about 500 µs was selected as we have previously shown that the ILD of the echo onset contains sufficient information to approach or avoid objects (e.g. Nguyen and Vanderelst, 2024, 2022; Mansour et al., 2019; Vanderelst et al., 2015). The ILD Δ*I*:
(5)

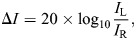
was converted to an estimate of the azimuth α_0_ of the origin of the first echo. This was done using a lookup table:
(6)

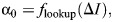
empirically determined by ensonifying a free-standing 15 cm diameter cardboard tube. Although the lookup table was obtained using a simple reflector that returns a single isolated echo, the leaves (with or without dragonflies) often return multiple overlapping echoes, depending on the relative position of the leaf and the robot. Therefore, the estimated azimuth might not be very accurate. Moreover, the exact azimuthal position of an extended reflector is not a well-defined concept because it occupies, by definition, a range of azimuthal positions. However, if the estimate α_0_ is a somewhat reliable indicator of whether the closest reflector can be found to the left or right of the sonar head, this allows approaching the leaves. The robot then performed one of three behaviors based on the estimated distance *d*_0_ and the estimated azimuth α_0_.

#### Random exploration

The random exploration (Fig. 4, label d) behavior was invoked if *d*_0_>100 cm (Fig. 4, label c). A distance larger than 100 cm indicated that none of the echoes crossed the threshold and, therefore, that the robot was not detecting an echo. If the random exploration was triggered, the sonar head assumed a new random position in the ranges:
(7)

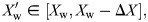

(8)

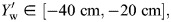

(9)

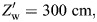

(10)

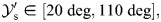

(11)

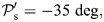
with *X*_w_ the current position *X* of the sonar head in the world coordinate frame and Δ*X*∈[2.5 cm, 10 cm]. This random jump corresponds to the randomization of the 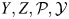 coordinates of the sonar head in the same ranges as at the beginning of the trial. In the *X* direction, the sonar head is shifted toward the negative end of the *X*-axis with respect to its current coordinate *X*_w_. This biased the random exploration behavior to explore along the row of leaves.

#### Pitch correction

If the estimated azimuth α_0_ was less than 5 deg (Fig. 4, label e), the robot performed pitch correction behavior (Fig. 4, label f). This behavior tried to adjust the sonar head's pitch 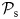 towards the origin of the echo.

In bats, it is believed that determining the vertical position of reflectors is primarily based on frequency- and direction-dependent filtering by the outer ears (Lawrence and Simmons, 1982; Aytekin et al., 2004; De Mey et al., 2008; Reijniers et al., 2010). However, the transfer function of our sonar sensor is circularly symmetric and operates with a narrowband signal centered around 42 kHz. As a result, it cannot estimate the vertical position of reflectors using the same approach. To address this limitation, we collected five measurements in pitch orientations 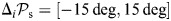 around the current pitch 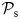. This process simulated the frequency-dependent sweep of the main lobe observed in the head-related transfer function of *M. microtis* (Vanderelst et al., 2010). In other words, this is an analogue of the bat's ability to extract elevation cues from a single echo using its head-related transfer function. We do not claim that this mimics real head-pitching behavior in the bat – only that both bat and robot can rely on coarse elevation cues to guide behavior. For each pitch, we calculated the sum Σ*I*_*i*_:
(12)

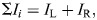
and obtained the pitch angle 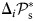 for which Σ*I*_*i*_ is the largest using spline interpolation. The pitch 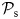 of the sonar head was updated as:
(13)

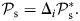
Geipel et al. (2019) observed that when *M. microtis* hovers approximately 10 cm in front of a leaf, it exhibits highly variable head pitch angles 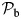 ranging from approximately 20 deg (downward) to −80 deg (upward), with a peak around −55 deg (see Fig. S1). At greater distances from the leaf, the bat assumes less variable and less extreme head pitch angles. Although there are no data documenting the bat's head aim angles for the range of distances modeled in our experiments (approximately 0.5 to 1.5 m), it appears that *M. microtis* adopts less extreme (but still negative) pitch angles as it moves further from the leaf. Based on this observation, we limited the pitch range of the sonar head to a minimum of −45 deg during our experiments. As shown in Fig. S2, this is a conservative limit. For the start of each trial, we selected an initial pitch angle of −35 deg for the sonar head.

If the new pitch value determined by Eqn 13 was less than −45 deg, the new sonar head pitch was set to 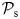
=−35 deg instead after the sonar head was moved to a new height above the floor, that is, in the direction of *Z*. The difference in *Z*_w_ (Δ*Z*_w_) is given by:
(14)

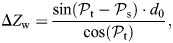
with 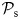 the current sonar head pitch and 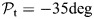. This keeps the sonar head pointing at the same point *o* (see Fig. 8) as before but with a pitch set to –35 deg. In sum, the pitch correction behavior attempted to adjust the sonar head pitch 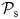 to face the origin of the first detected echo (at a distance *d*_0_). If the pitch angle became too large, the sonar head was moved upward in the *Z* direction to compensate for this. In other words, if the robotic bat must tilt its head sharply upward to keep pointing at the strongest echo, it moves up so that the head can remain at a shallower angle.

At this point, the robot infers the location *X*_0_,*Y*_0_,*Z*_0_ from which the first echo appeared to come based on its own pose and the estimated distance and azimuth:
(15)

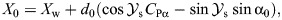

(16)

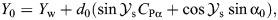

(17)

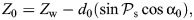
with 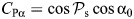.The robot then compares (Fig. 4, label g) this position against the locations from which it previously captured prey (see next section), if any. If the position *X*_0_,*Y*_0_,*Z*_0_ was closer than 10 cm (*D*_min_<10 cm; Fig. 4, label g) to one of these previously recorded positions, the random exploration behavior is triggered (Fig. 4, label d). This mechanism is a simple mechanism that was added to ensure that the robot continues to move along the track and does not return to the position that it was previously, perhaps erroneously, stopped at. It models the assumption that if a bat has erroneously tried to pick prey from a leaf, it will not revisit the very close neighborhood of the position at which it was disappointed.

#### Echo following

If the estimated distance *d*_0_ was greater than 50 cm and none of the other behaviors were invoked (Fig. 4, label h), the echo following behavior was invoked (Fig. 4, label i). The sonar head's yaw 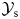 was updated proportionally to the estimated α_0_:
(18)

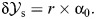
With *r*=0.75, after correcting the yaw 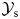, the robot moved forward 2.5 cm. Data collected by Geipel et al. (2019) indicate that at distances less than 30 cm from a leaf, *M. microtis* typically moves approximately 0.5 cm between calls (Fig. S5). Because no data are available for greater distances, we set the robot's displacement between calls to 2.5 cm, which corresponds to 1.25 cm when corrected for scaling the setup by a factor of 2. This adjustment causes the robot to move faster than the bat, at least at short distances from the leaves, thereby speeding up experimentation.

The robot's movement was directed along the updated orientation of the sonar head's *X*_s_-axis, projected onto the world's horizontal plane. In other words, the sonar head moved forward without changing its height above the ground. In summary, the echo following behavior aimed to adjust the sonar head's orientation to face the source of the first echo and move in that direction.

#### Finding leaves and end of trial

If the estimated distance *d*_0_ was less than 50 cm (Fig. 4, label h), the robot control program paused, assuming that the robot had made a capture attempt (Fig. 4, label j). The distance of 50 cm was dictated by the duration of the sonar emission; approaching targets closer resulted in forward masking of the echoes. The robot's emissions had a fixed duration of approximately 2 ms, whereas *M. microtis*, when gleaning prey from leaves, uses calls with a duration of only 0.2 ms (Geipel et al., 2013).

Importantly, and as said earlier, this stopping distance still allowed us to model the relevant decision-making distances for the bat. Because the setup was scaled by a factor of 2, the stopping distance of 50 cm corresponds to 25 cm in the original bat scale. This aligns with the findings of Geipel et al. (2019), who documented that *M. microtis* begins its final approach and makes a decision about which leaf to target at distances shorter than approximately 25 cm (see Fig. S2).

Pausing the robot's program allowed us to evaluate whether the robot had stopped in front of a leaf and, if so, whether a dragonfly occupied the leaf. The robot was considered to have stopped in front of a leaf if the gaze of the sonar head, visualized by the embedded laser diode, intersected the leaf.

If a dragonfly was present on the leaf that was focused on by the sonar head, it was removed to simulate a successful capture of the prey. This mimicked the natural scenario in which a bat leaves a leaf empty of prey after visiting it. Irrespective of whether a prey item was present, the location *X*_0_,*Y*_0_,*Z*_0_ (Eqn 17) is stored in memory (Fig. 4, label k). The experimenter then resumed the program, and the trial continued. Upon resuming, the robot performed a random exploration step. An experimental trial ended when the sonar head reached the end of the track (*X*_w_<–20 cm; Fig. 4, label l).

### Conditions and trials

We evaluated the algorithm described above using two different experimental setups. In the first setup, we tested six different tilt angles for the leaves. The leaf used by Geipel et al. (2019) was tilted backward by approximately 23 deg (=–23 deg tilt angle). To assess whether our sensorimotor strategy would generalize to other leaf tilts, the leaves in our experiments could have tilt angles of 10, 0, –10, –20, –30 or –40 deg. For each tilt angle condition, we placed the dragonfly on each of the five leaves for five iterations, resulting in a total of 25 trials per condition.

In the second setup, we introduced stylized artificial leaves and a simplified dragonfly target to further test the robustness of the sensorimotor model. For the second setup, we focused on a single leaf tilt angle of –30 deg. This setup was also used for trials without the dragonfly, where the robot's behavior without a prey target was evaluated.

### Digitizing the setup

After completing the trials for each leaf pitch angle condition, we digitized the first setup using an Artec Leo 3D scanner (Artec, Santa Clara, CA, USA). The setup was scanned with and without dragonflies on each leaf. These scans produced large point clouds that were subsampled using open-source Meshlab software (Cignoni et al., 2008). Meshlab was also used to calculate a normal vector for each point in the point cloud based on its nearest neighbors. The coordinates of the point clouds were registered with the world frame axes (*X*,*Y*,*Z*) using a reference object placed in the setup during the 3D scanning process.

The registered 3D point clouds and the normals at each point enabled us to calculate the incidence angle for each sonar measurement taken by the robot. For each measurement, we determined whether the sonar head's acoustic beam intersected a leaf by projecting an imaginary cone with a 15 deg opening angle along the sonar head's *X*_s_-axis. The closest point on a leaf within this cone was considered the intersection point of the acoustic beam and the leaf. If no point was found, the acoustic beam was deemed not to intersect a leaf.

The angle of incidence η was calculated as the angle between the normal at the intersection point and the *X*_s_-axis of the sonar head. For leaves with a dragonfly, calculations were performed using the 3D scan containing the dragonfly. For leaves without a dragonfly, the corresponding 3D scan without a dragonfly was used.

## RESULTS

We addressed the following questions to test our claim that the sensorimotor model performs efficient foraging by exploiting the SRE without determining the position and orientation of each leaf. First, we assessed the robot's success rate in finding the leaf with the artificial dragonfly. To this end, we determined how often the robot stopped while focusing on a leaf with and without a dragonfly. Second, we tested whether the robot's behavior is consistent with the SRE-based strategy proposed by Geipel et al. (2013, 2019). This was done by analyzing both the echo amplitudes experienced by the robot and the incidence angles η attained by the robot. Third, we addressed the efficiency of the proposed strategy by comparing the effort spent ensonifying leaves with and without dragonflies. Fourth, we tested the strategy's robustness and generalizability by assessing its success rate in the second setup, where the leaves overlap and the leaves and dragonfly are stylized.

### Success rate

**Fig. 5.**
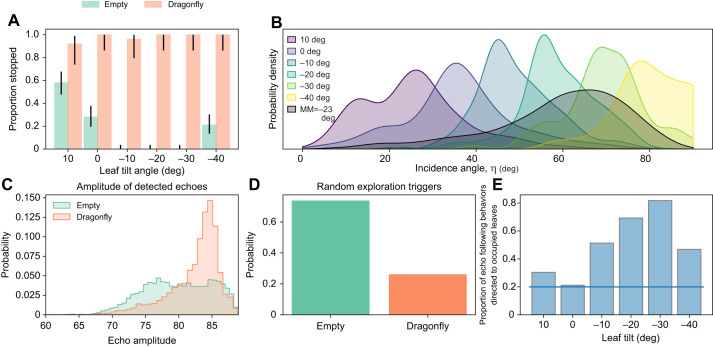
**Results for the first experimental setup.** (A) The proportion of leaves with and without a dragonfly in front of which the robot stopped (Fig. 4, label j) in the first of the two robotic setups. For leaves with a dragonfly, this graph shows the capture rate. For leaves without a dragonfly, the graph shows how often the robot mistook an empty leaf for one with insect prey. The vertical lines show 95% confidence intervals. (B) The distribution of the incidence angles (η) as a function of the leaf tilt (depicted in different colors). The empirical incidence angles observed by Geipel et al. (2019) in *Micronycteris microtis* are also shown (labeled MM). Note that in these experiments, the leaf tilt was approximately −23 deg. (C–E) Acoustic cues used by the robot. (C) Distribution of echo amplitude for calls in which the robot's line of view intersected with leaves with and without a 3D-printed dragonfly. (D) The proportion of times the random exploration behavior (Fig. 4, label d) was triggered because echoes did not cross the amplitude threshold for calls in which the robot's line of view intersected with leaves with and without a 3D-printed dragonfly. (E) The proportion of echo following behaviors directed at occupied leaves as a function of leaf tilt. The horizontal line shows the proportion of calls that would be directed at an occupied leaf if the robot randomly ensonified the leaves (*P*=0.2).

Fig. 5A shows the probability that the robot stopped in front of a leaf as a function of the state of the leaf (empty or occupied by the artificial dragonfly) and its tilt angle in the first of our two setups. Across the leaf tilt angles, the robot stopped in front of 18% (107 out of 600=25 trials×6 tilt angles×4 empty leaves per trial) of empty leaves. It stopped in front of 98% (147 out of 150=25 trials×6 tilt angles×1 leaf occupied per trial) of leaves occupied by the 3D-printed dragonfly. In addition, the robot stopped several times when not positioned in front of a leaf. This happened only when the leaves were tilted at angles of 10 deg (6 stops) and 0 deg (4 stops).

The results shown in Fig. 5A confirm that the robot could successfully approach leaves with insects (while mostly ignoring empty leaves). In particular, Fig. 5A shows that the successful discrimination between empty and occupied leaves depended on the tilt angle of the leaf. The robot successfully ignored all empty leaves for tilt angles of −10, −20 or −30 deg. For leaves tilted by 10, 0 and −40 deg, the robot sometimes erroneously stopped in front of an empty leaf. The error rate was highest when the leaves were tilted forward by 10 deg.

### Use of the SRE

As mentioned, the sensorimotor model that controls the robot (Fig. 4) was designed to take advantage of SRE. However, to confirm whether the robot made actual use of SRE, we evaluated the incidence angles attained by the robot. Note that the robot did not control these incidence angles directly. They occur as a function of the relative poses (position and orientation) of the robot and the leaves as the robot executes the sensorimotor loop. In addition to the angles of incidence, we also evaluated the echo amplitude experienced by the robot, as this was the acoustic cue that caused the robot to abandon the echo following behavior and return to the random exploration behavior (Fig. 4, label d).

Fig. 5B shows the incidence angles at which the robot ensonified the leaves as a function of the leaf tilt angle in the first setup. The graphs also include the incidence angles observed in *M. microtis* by Geipel et al. (2019). In particular, the incidence angles used by *M. microtis* in the experiments reported by Geipel et al. (2019) closely align with those of the robot when the leaves were tilted at −20 and −30 deg. Interestingly, in the behavioral experiments by Geipel et al. (2019), the leaf tilt (∼–23 deg) fell in this range.

Geipel et al. (2013, 2019) showed that larger incidence angles result in lower echo amplitudes for empty leaves compared to leaves with a prey item due to the SRE. Fig. 5C shows the distribution of the observed echo amplitudes. The distributions in Fig. 5C confirm that, in our experiments, leaves with the artificial dragonfly tended to return louder echoes.

The robot used the echo amplitude to decide whether to abandon the echo following and return to random exploration (Fig. 4, label d) or not. Fig. 5D shows the probability that the random exploration behavior is triggered by a low echo amplitude. This graph shows that random exploration was approximately four times more likely to occur when the robot looked at an empty leaf than when it looked at a leaf with a prey item on it.

**Fig. 6.**
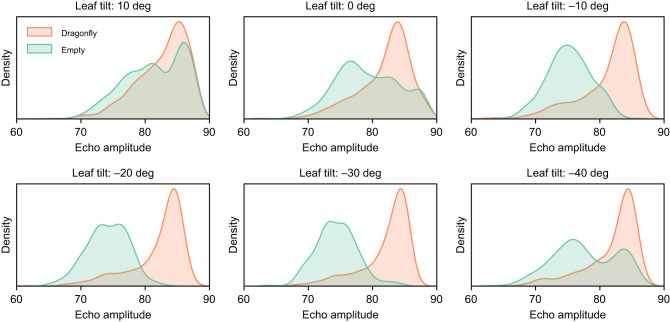
**The distribution of echo amplitude for calls directed towards empty and occupied leaves as a function of leaf tilt angle in the first setup.** This plot separates the data shown in Fig. 5C for different left tilt angles.

We further analyzed the robot's use of the echo-amplitude cue. Fig. 6 shows the distribution of echo amplitudes as a function of the leaf tilt angle for calls directed at empty and occupied leaves. The graph reveals that the echo amplitude is more effectively distinguished between leaves with and without dragonflies at tilt angles of −10, −20 and −30 deg. In contrast, for other tilt angles, the amplitude distributions for occupied and empty leaves overlapped more, reducing their discriminative value. Thus, Fig. 6 explains why the robot was more successful in locating leaves with a dragonfly based on echo amplitude when the leaves were tilted at −10, −20 or −30 deg.

Figs 5C and 6 show that for a single, randomly sampled view of an occupied leaf, the echoes are somewhat more likely to be louder than for an unoccupied leaf. However, the behavior of the robot amplifies the difference in echo amplitude through a positive feedback mechanism. Upon detecting a strong enough echo, the robot will shift its focus to a direction from which the echo seems to come. Hence, the next echo is likely to be louder still. Additionally, the robot approaches the leaf, which also produces louder echoes. For empty leaves, this positive feedback is absent. Even if they return an above-threshold echo, because the echoes are specular, the next echo, measured from a different vantage point, could be very weak. So, although Figs 5C and 6 show that randomly selected echoes do not necessarily fall into two separable intensity categories corresponding to empty versus occupied leaves, the way the robot samples (and subsequent samples are not independent when the leaf is occupied) leads to stable echoes for occupied leaves and unstable ones for empty leaves. The model therefore exploits the temporal dynamics of echo sampling: occupied leaves produce stable, increasing amplitude feedback, whereas specular empty leaves generate transient peaks that quickly vanish as the robot moves.

In summary, from the results shown in Figs 5C,D and 6, we conclude that the robot exploited the SRE despite not explicitly localizing the leaf or inferring its tilt angle.

### Efficiency

We conjectured that a strategy that avoids determining a leaf's position and orientation from an echo train would be more efficient than one that does. To our knowledge, there are no data on how many calls *M. microtis* would need to make such deductions, preventing a direct calculation of the efficiency gain from our proposed strategy. However, we can evaluate the efficiency of the strategy by comparing its performance across different leaf tilt angles. Hence, we measure the efficiency of the proposed sensorimotor model by assessing how much time it spends approaching leaves occupied with insects compared with leaves without prey. A maximally efficient strategy would not spend time approaching empty leaves while focusing all sensory effort on leaves with potential prey. Additionally, we can compare the robot's efficiency with a strategy in which leaves are randomly ensonified.

Fig. 5E shows the probability that the robot executed an echo following behavior (Fig. 4, label i) directed at a leaf with an artificial dragonfly (i.e. the robot emission is directed at an occupied leaf). The graph also shows the expected proportion of echo following behaviors to occupied leaves if the robot would direct its echo following behavior at random leaves (i.e. *P*=0.2). This graph shows that for leaf tilts of 10 deg, there was only a slight bias for the robot to approach the occupied leaves. For leaves tilted by 0 deg, there was no bias. However, for leaf tilts −10, −20 and −30 deg, we found that approximately 50–80% of the approaches were directed at occupied leaves. This is between 2.5 and 4 times as many as expected by chance alone. When the leaves were tilted −40 deg, this proportion decreased to approximately 50% again (or approximately 2.5 times what would be expected by chance).

### Generalizability

We conducted 45 trials using the second setup, as shown in Fig. 3. The stylized dragonfly was placed 15 times on each of the three leaves. The robot successfully stopped in front of leaves 1 and 2 in all 15 trials. For leaf 3, it failed to stop correctly in one out of the 15 trials. Overall, the robot stopped successfully in front of the correct leaf in 44 of 45 trials. However, it mistakenly stopped in front of empty leaves twice: once in front of leaf 2 and once in front of leaf 3. To confirm that the robot does not respond to empty leaves, we conducted 15 additional trials without placing the dragonfly on any leaf. In these trials, the robot passed by all the leaves and did not stop in front of any of them.

## DISCUSSION

In ensonification experiments, Geipel et al. (2019) demonstrated that the echo intensity can be used to discriminate between leaves with and without perched insect prey when ensonifying the leaves at sufficiently large incidence angles. They termed the hypothesis that *M. microtis* uses this effect to find prey the SRE hypothesis. Furthermore, their experiments showed that the behavior of the bat was in agreement with the predictions of the SRE hypothesis: the bats ensonified the leaves in the expected range of incidence angles. However, the authors did not address the specific mechanisms or behaviors that would allow *M. microtis* to exploit SRE.

The present study presents a sensorimotor mechanism that exploits the SRE to perform effective and efficient foraging. We used a methodology known as biorobotics (Webb, 2001) or robotics-inspired biology (Gravish and Lauder, 2018) to test our hypothesis. In contrast to a computer model, this approach does not require modeling the interaction of sound with complex environments. In fact, modeling the signals the organism receives tends to be the major challenge faced by computer models (Marr, 1982). Usually, this challenge is addressed by introducing substantial simplifications (Beckers et al., 1994; Lambrinos et al., 2000; Webb, 2001). As an alternative to computer models, many researchers have used robots to test perceptual theories (for reviews, see Webb, 2008, 2009, 2001; Carmena, 2002; Long, 2012; Gravish and Lauder, 2018). In contrast to simulations, robotic models, such as ours, do not (need to) make simplifying assumptions about the complex world and its signals.

The results of our robotic experiment confirm that a strategy that relies solely on echo intensity and ILD as cues enables the robot to distinguish and approach leaves with prey. In the first setup, for leaves with tilt angles of −10, −20 and −30 deg, the discrimination between empty and occupied leaves was nearly perfect. For leaves tilted 10, 0 and −40 deg, discrimination was less successful as more empty leaves were mistaken for prey-occupied ones (Fig. 2A).

Discrimination was most successful for leaf tilts that resulted in large, but not extreme, ensonification incidence angles (Fig. 2B). At these angles, the difference in echo amplitude distributions between prey-occupied and empty leaves was greatest (Fig. 6). However, at very large incidence angles, the difference in echo amplitude decreased, probably because diffraction around the edges of the leaf becomes more prominent. Hence, our results show that the proposed sensorimotor model is capable of exploiting the SRE to successfully discriminate between empty and occupied leaves with tilt angles ranging from −10 to −30 deg without explicitly localizing these leaves or explicitly controlling for the incidence angles. Instead, the effectiveness of the model depends on the temporal dynamics of echo sampling: occupied leaves produce stable amplitude feedback, whereas specular empty leaves generate transient peaks that quickly vanish as the robot moves.

By analyzing the distribution of the echo amplitudes and the ensonification incidence angles, we conclude that the robot's success stemmed from using the SRE to discriminate between leaves with and without prey. For leaf tilt angles between −10 and −30 deg, where discrimination was most successful, the robot achieved incidence angles broadly within the range used by the bat (Fig. 5B), which in turn correspond to the optimal incidence angles as found in an ensonification study (Geipel et al., 2019). As a consequence, the echoes received from the occupied leaves were louder (Figs 5D and 6), and the approach was less likely to be aborted by the robot (Fig. 5D).

For leaf tilt angles between −10 and −30 deg, the sensorimotor model was not only successful but also efficient. Within this range, the model directed up to four times as many calls toward occupied leaves as would be expected from a random strategy (Fig. 5E). However, the strategy's efficiency decreased for tilt angles outside this range.

**Fig. 7.**
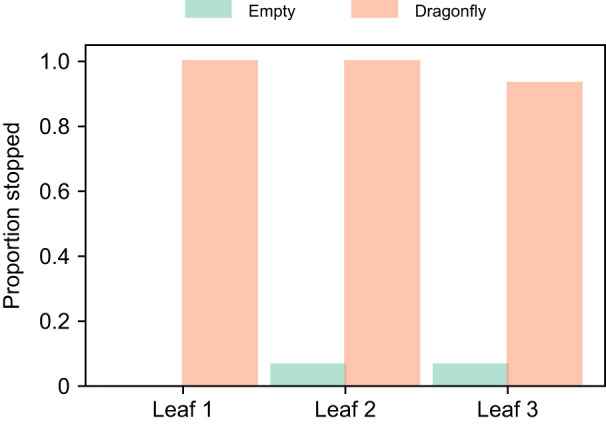
**Success rates in the second setup with closely spaced stylized leaves and a stylized dragonfly.** For each of the three leaves, 15 trials were conducted. The bars show the proportion of trials in which the robot stopped in front of each leaf, either when it was empty or when the dragonfly was present.

We also tested whether the results depended on the spacing and shape of the leaves (as well as the dragonfly) by running additional trials using a second setup. In this setup, we focused solely on leaves with a tilt angle of −30 deg. The robot's performance was comparable to that in the first setup (compare Fig. 5A for −30 deg and Fig. 7). When the leaves overlapped, the robot mistakenly stopped twice in front of an empty leaf out of 45 trials and missed stopping in front of one dragonfly. This corresponds to a success rate of more than 97% and a false positive rate of approximately 4%.

Taken together, the success and efficiency measures suggest that the proposed sensorimotor model represents a plausible strategy to guide *M. microtis* successfully and efficiently toward prey-occupied leaves. Importantly, to implement this strategy, the bat, like the robot, can rely on simple amplitude cues and a straightforward decision process. This shows that the apparent intelligence required to execute the strategy largely emerges from the interaction dynamics between the sensorimotor model and the environment. Thus, the proposed strategy can be viewed as the behavioral equivalent of a sensory-matched filter, which selectively responds to environmental information (Wehner, 1987) to energetically optimize the execution of the prey-finding task (Warrant, 2016). However, as we explain in the next section, this strategy is only effective if certain environmental geometric constraint conditions are met and/or matched by the behavior.

### Interpreting bat behavior: geometric constraints

In the following, we discuss how the geometric and acoustic constraints formalized in our model explain the observed foraging behavior of *M. microtis* and may generalize to other substrate-gleaning bats. In addition, this geometric interpretation will be useful in deriving testable predictions from the model.

**Fig. 8.**
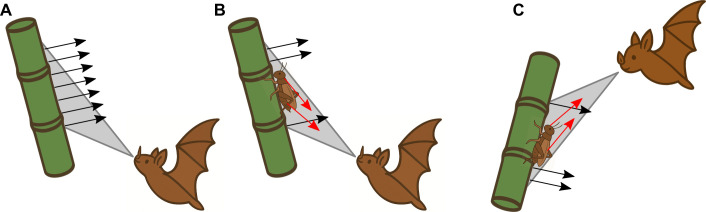
**Illustration of the geometric constraints required for exploiting the SRE.** (A) A bat ensonifying a bamboo stem. The surface normals (in the cross-sectional plane defined by the bat and the bamboo stem) are shown as parallel black arrows, as the surface of the stem is assumed to be flat. In this configuration, none of the normals within the projected area point back towards the bat. This means no strong echoes are returned to the bat. Acoustic phenomena beyond the scope of ray acoustics may still produce weak echoes. (B) The same bamboo stem with prey sitting on it. The complex shape of the prey introduces additional normals, some of which point back to the bat (drawn using red arrows). This configuration results in strong echoes being returned to the bat, enabling detection of the prey. (C) It should be noted that the same geometric reasoning also indicates that the situation is symmetric. In our experiments, we modeled bats looking upward at negatively tilted leaves because this is the orientation the bat assumed in the experiments reported by Geipel (2007) and Geipel et al. (2013, 2019). However, if the surface were instead tilted forward (positive tilt), the same oblique incidence angles could be obtained if the bat were looking downward. Thus, the mechanism is not inherently limited to negative tilt angles, but the tilt of the leaves and the bat's gaze direction (behavior) must align to create the required oblique geometry.

To illustrate the geometric constraints required for exploiting the SRE, regardless of the particular prey or substrate, we used a cricket sitting on a bamboo stem in Fig. 8. These geometric constraints can be stated most simply by assuming that ray acoustics apply. This is a reasonable assumption for bats that use ultrasonic calls and substrates with realistically large dimensions, although it remains a simplification. A second helpful simplification is to model the bat's emission directionality as a sharply delineated cone. Under these assumptions, the bat can be expected to receive detectable echoes from an extended substrate if some of the substrate's normals that fall within the emission cone point back to the bat.

In Fig. 8A, the bat is depicted ensonifying a section of a bamboo stem. The surface normals (in the cross-section plane defined by the bat and the bamboo stem) are drawn and, in this configuration, are all parallel because we neglect the curvature of the bamboo stem and assume the surface to be flat in this plane. We also have drawn the intersection of the bat's emission cone with the substrate in grey. None of the normals in this area point back to the bat. Consequently, assuming ray acoustics, the bat would not receive an echo from the bamboo. In reality, acoustic phenomena not captured by ray acoustics and deviations from complete flatness might still result in a weak echo. In Fig. 8B, prey sits on the bamboo. The complex shape of the prey introduces non-parallel local normals within the intersection of the bat's emission cone. Some of these will point towards the bat (illustrated by one normal drawn in red), resulting in strong echoes. Again, although this simplification captures the most important acoustic aspects, it is not complete. For example, reflections between the prey and the smooth surface might further enhance the echo's detectability (Siemers et al., 2005). Finally, it should be noted that, an equivalent, symmetric geometric condition to the one depicted in Fig. 8B exists: instead of looking up at a backward tilted surface (i.e. negative tilt angles in the coordinate system used in the present study), the bat could look down onto forward tilted surfaces (Fig. 8C). However, in previous experiments (Geipel, 2007; Geipel et al., 2013, 2019), the bats approach prey from below, looking up. Therefore, this is the geometric situation modeled in the present study.

This geometrical analysis, based on simplified acoustics, shows that for the bat to exploit the SRE, it needs to position itself such that the normals of the ensonified part of the substrate fall outside its emission cone. As can be seen in Fig. 8, this is equivalent to stating that the incidence angle should be sufficiently large. Our robotic experiments show that, under these conditions, a simple amplitude-based algorithm can be an efficient and effective way to find prey.

The incidence angle is determined by (1) the height of the ensonified part of the substrate above the bat's flight path, (2) its horizontal distance from the flight path and (3) its tilt angle. This allows the bat to adapt its flight path to local conditions, i.e. the local distribution of leaf (or other substrate) tilt angles, when hunting in a known environment. The assumption that *M. microtis* has knowledge about the distribution of leaf orientations (but not each individual leaf orientation) on which it is foraging is not implausible. Albrecht et al. (2007) found *M. microtis* using foraging areas of approximately 120×120 m. Within this foraging range, the core area used was much smaller (approximately 30×30 m). Hence, as documented in other species of bat (for references, see Schnitzler and Denzinger, 2024), *M. microtis* shows considerable site fidelity.

### Testable predictions

Before concluding, we outline three testable predictions that can be derived from the proposed sensorimotor model.

#### Weak echoes terminate leaf approach

The rationale underlying the model is that approaches to empty leaves can be abandoned when the returning echo becomes too weak. This predicts that a bat's decision to abort an approach is triggered by the detection of such a weak echo. The prediction extends beyond *M. microtis* to any bat species that gleans prey from smooth, specularly reflecting substrates. More generally, it can be expressed in terms of the geometric constraints outlined above: we predict that bats will abandon an approach when the surface normals of the target fall outside the emission cone, causing reflected energy to miss the bat and resulting in a sufficiently weak echo.

This prediction could be tested by assessing the echoes received by bats as they forage. One approach is to equip bats with on-board microphones (e.g. Greif and Yovel, 2019; Mantani et al., 2012), allowing direct measurement of returning echoes. Alternatively, if bat position and orientation can be tracked during approach, the expected echo strength could be approximated by acoustic simulation or geometric modeling.

#### Sonar aim reflects statistical knowledge of local substrate geometry

The model predicts that bats do not adjust their incidence angle on a leaf-by-leaf basis; indeed, we assume that *M. microtis* does not infer the position or orientation of individual leaves. Instead, we propose that it adopts a sonar aim orientation and flight path that match the typical tilt of leaves in its local environment. As mentioned above, some bats (Schnitzler and Denzinger, 2024), including *M. microtis* (Albrecht et al., 2007), exhibit considerable site fidelity, which would allow them to learn and adjust their sensorimotor strategy to local geometric conditions. As a corollary, we also predict that substrate-gleaning bats should generally show high site fidelity and that some degree of learning would be required to optimize foraging efficiency when encountering a novel environment.

This prediction could be tested by tracking the sonar aim and foraging performance of bats in environments with different dominant leaf orientations, i.e. extending the experiments conducted by Geipel et al. (2019). If bats rely on statistical knowledge of local geometry, sonar aim should remain stable within a given environment but shift across environments. Furthermore, exposing bats to a new environment with unfamiliar (or altered) leaf tilt distributions should initially reduce foraging efficiency, followed by gradual improvement as bats adapt their aim and flight paths to the new geometry.

#### Robustness against prey and leaf shapes

The model predicts that successful prey detection does not depend on the detailed shape of the leaves or prey, but rather on their acoustic scattering properties. Because the decision to continue or abandon an approach relies solely on echo amplitude, the system should generalize across a range of natural and artificial leaf and prey shapes, provided that occupied leaves return consistently detectable echoes. This prediction is supported by our robotic experiments, in which the sensorimotor strategy remained effective despite the use of stylized leaves and simplified prey items.

Geipel (2007) presented *M. microtis* with various natural and artificial prey items, including a dragonfly shape made of aluminium foil. Although there was some variability in the proportion of targets taken by the bats, all but the paper dragonfly were taken to some extent. Unfortunately, it is unknown how these targets differed in the echo strength they produced. Extending such experiments, including systematic variation of both prey and substrate shapes while measuring or controlling for echo strength, would help clarify the extent to which gleaning bats are insensitive to the detailed shapes of prey and substrates, provided the echo remains consistently loud enough.

### Conclusion

In conclusion, by showing that the proposed sensorimotor model can exploit the SRE to efficiently and effectively find prey, our results increase the plausibility of *M. microtis* actually using the SRE under natural conditions. In addition, the proposed mechanism, as implemented in a robotic echolocating agent, makes specific predictions about the relationship between bat flight path parameters and leaf orientation distributions that can be used to further test the SRE hypothesis.

## Supplementary Material

10.1242/jexbio.250818_sup1 Supplementary information
