# Supplementary material for: A robotic model of efficient prey finding in the gleaning bat Micronycteris microtis
Source: J Exp Biol. 2026 Jan 14;229(1):jeb250818. doi: 10.1242/jeb.250818 (PMC12848576; doi:10.1242/jeb.250818)
Supplement: Supplementary information [file jexbio-229-250818-s1.pdf]

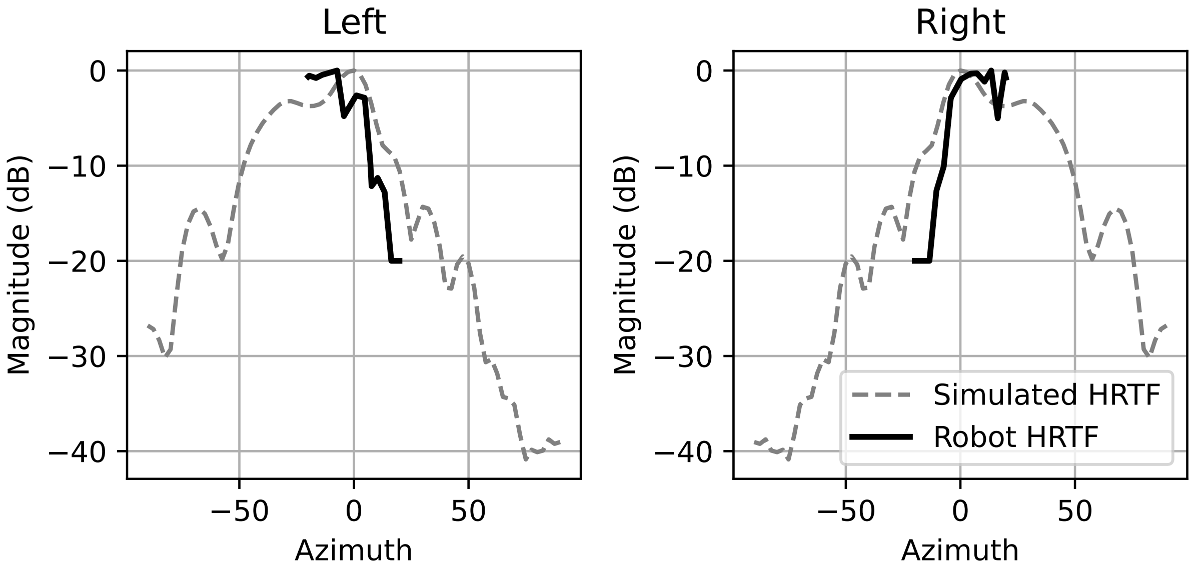

Fig. S1. Graph comparing the simulated HRTF of *M. microtis* with that of the robot. The simulated data is for the left ear, convolved with the emission directionality, of *M. microtis* as described by Vanderelst et al. (2010). The data shown are for a band around 100 KHz (95 to 105 kHz) and averaged across ±10 degrees in elevation. The robot data is only shown for ±20 degrees in elevation as, beyond this range no echoes crossed the threshold used for echo detection. The target for collecting these data was a freestanding 15 cm diameter cardboard tube. The echo was detected and integrated using the same approach as during the experiments.

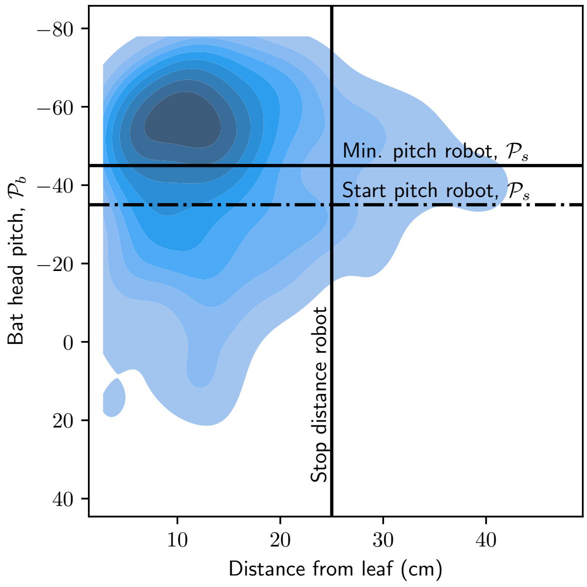

Fig. S2. Data from Geipel et al. (2019) showing the distribution of the vertical head aim angles of the bat (corresponding to the pitch $\mathcal{P}_s$ in our robot) as a function of the distance from the leaf. Negative angles mean that the bat is looking up. The full horizontal line indicates the minimum value for $\mathcal{P}_s$ we allowed the robot to assume in our experiments, i.e., $\mathcal{P}_s = -45°$. The dashed horizontal line indicates the initial pitch angle of the robot, $\mathcal{P}_s = -35°$. This is the pitch angle assumed by the robot at the beginning of the trial and after invoking the *Random Exploration* behavior. The vertical line shows the distance to the leaf at which the robot stopped, i.e., 25 cm. If the estimated distance $d_0 < 50$, the robot stopped (note that the robotic setup was scaled by a factor of 2 to compensate for the wavelengths used by the robotic sonar sensors).

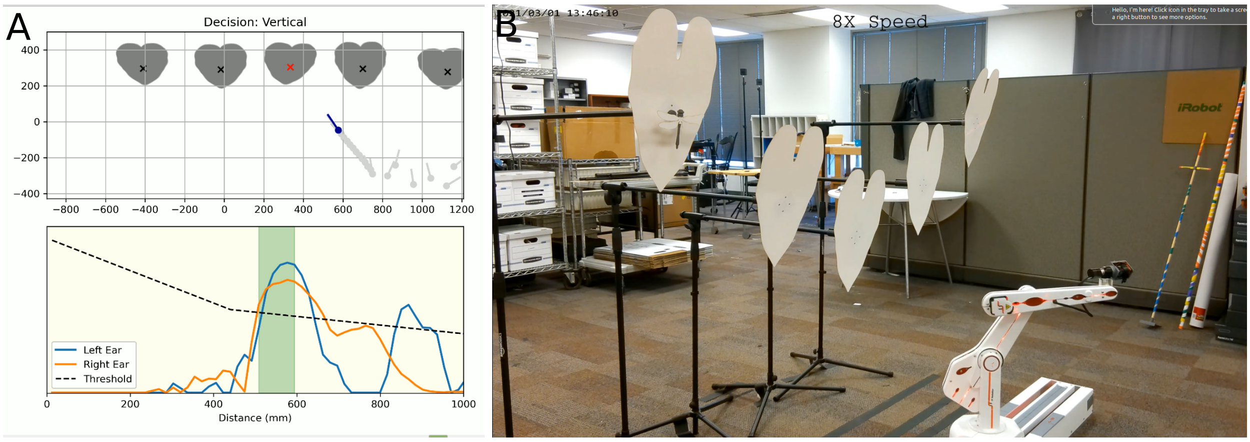

Fig. S3. Screenshots of the video provided as supplementary material. (A) A still from the videos provided in the supple-mentary material. Top panel: A top-down view of the setup. The outlines of the leaves are shown in gray. Black crosses mark the center positions of the leaves, where dragonflies are placed, while the red cross indicates the current position of the dragonfly. The position and orientation of the sonar head are represented by the blue marker. Past positions of the dragonfly are shown in gray. Bottom panel: The echoes received at the left and right ears are displayed, along with the threshold level and integration window. These are available in the accompanying repository in the `Approach Videos` folder. (B) A screenshot of one of the videos showing selected trials. These are available in the accompanying repository in the `Demo Videos` folder.

Journal of Experimental Biology • Supplementary information

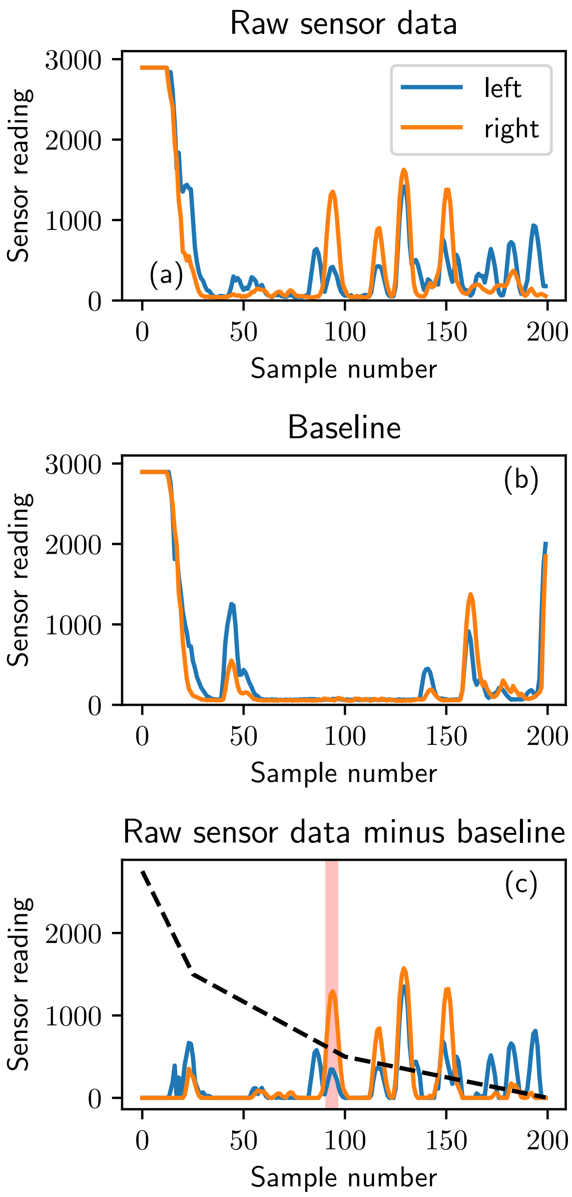

Fig. S4. Illustration of the echo processing. (a) Raw data from the left and right sonar sensors. (b) Baseline data obtained by pulsing the sensors in the absence of a reflector. The data in panel (b) are subtracted from the raw data to obtain the data depicted in panel (c). The black line in panel (c) is the threshold that an echo needs to cross before it is considered strong enough to come from a leaf. The first data point that crosses the threshold is considered the onset of the echo, and a short interval (shaded in red) following this onset is integrated to obtain the echo strength in the left and right sensor (ear). The y-axes represent arbitrary units as provided by the sonar system's analog-to-digital converter. These values correspond to the envelope (amplitude) of the underlying 42 kHz carrier wave.

Journal of Experimental Biology • Supplementary information

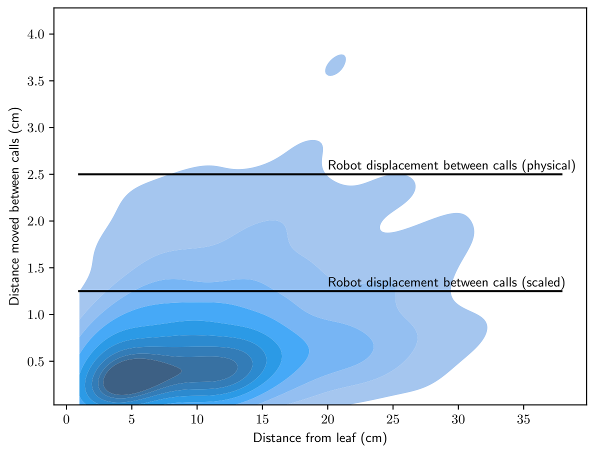

Fig. S5. Data from Geipel et al. (2019) showing the distribution of the bat's displacement between calls as a function of the distance from the leaf. At distances less than 30 centimeters from the leaf, the bat's displacement between calls is mostly below 1 cm.

References

Inga Geipel, Jan Steckel, Marco Tschapka, Dieter Vanderelst, Hans-Ulrich Schnitzler, Elisabeth K.V. Kalko, Herbert Peremans, and Ralph Simon. Bats Actively Use Leaves as Specular Reflectors to Detect Acoustically Camouflaged Prey. *Current Biology*, 29(16):2731–2736.e3, August 2019. ISSN 09609822. doi: 10.1016/j.cub.2019.06.076.

Dieter Vanderelst, Fons De Mey, Herbert Peremans, Inga Geipel, Elisabeth Kalko, and Uwe Firzlaff. What noseleaves do for fm bats depends on their degree of sensorial specialization. *PloS one*, 5(8):e11893, 2010.

Journal of Experimental Biology • Supplementary information
